# Mental Fatigue and Exercise

**Published:** 1898-02

**Authors:** 


### Mental Fatigue and Exercise.

The *Journal of the American Medical Association* (Dec. 19th, 1896) quotes from the *Therap. Woch.*, that it has been generally accepted that muscular exercise, gymnastics, was beneficial after continued mental effort, and school children are often put through a course of gymnastics to dissipate their mental fatigue after the morning's study. But Bum and others now declare that recent tests have been demonstrated that fatigue from mental exertion is increased by muscular exertion, and that nothing will dissipate this fatigue but physical and mental rest, viz., sleep.

JAPANESE women are agitating the question of suffrage, are about to erect a women's university, and many of them are already practicing medicine.

